# The role of RNA m^5^C modification in cancer metastasis

**DOI:** 10.7150/ijbs.61439

**Published:** 2021-08-02

**Authors:** Qiaofeng Zhang, Furong Liu, Wei Chen, Hongrui Miao, Huifang Liang, Zhibin Liao, Zhanguo Zhang, Bixiang Zhang

**Affiliations:** 1 Hepatic Surgery Center, Tongji Hospital, Tongji Medical College, Huazhong University of Science and Technology, Wuhan, Hubei 430030, China.; 2 Hubei Province for the Clinical Medicine Research Center of Hepatic Surgery, Wuhan, Hubei 430030, China.; 3 Hubei key laboratory of Hepato-Pancreato-Biliary Diseases, Tongji Hospital, Tongji Medical College, Huazhong University of Science and Technology, Wuhan, Hubei 430030, China.

**Keywords:** Metastasis, cell migration, 5-methylcytosine, ribosome acid, regulatory network

## Abstract

Epigenetic modification plays a crucial regulatory role in the biological processes of eukaryotic cells. The recent characterization of DNA and RNA methylation is still ongoing. Tumor metastasis has long been an unconquerable feature in the fight against cancer. As an inevitable component of the epigenetic regulatory network, 5-methylcytosine is associated with multifarious cellular processes and systemic diseases, including cell migration and cancer metastasis. Recently, gratifying progress has been achieved in determining the molecular interactions between m^5^C writers (DNMTs and NSUNs), demethylases (TETs), readers (YTHDF2, ALYREF and YBX1) and RNAs. However, the underlying mechanism of RNA m^5^C methylation in cell mobility and metastasis remains unclear. The functions of m^5^C writers and readers are believed to regulate gene expression at the post-transcription level and are involved in cellular metabolism and movement. In this review, we emphatically summarize the recent updates on m^5^C components and related regulatory networks. The content will be focused on writers and readers of the RNA m^5^C modification and potential mechanisms in diseases. We will discuss relevant upstream and downstream interacting molecules and their associations with cell migration and metastasis.

## Background

In eukaryotes, a wide range of nucleotide modifications, including acetylation, methylation and glycosylation, are essential for cellular biological processes and carcinogenesis. Among the hundreds of chemical modifications, 5-methylcytosine (m^5^C) is a highly focused epigenetic modification that has been identified in DNA and various RNA families. 5-Methylcytosine in DNA (5mC) is well established as a regulator of gene expression and genome stability 1, 2. Otherwise, the m^5^C modification is also present in transcript. The biological roles of this modification in RNA, including RNA export, translation, RNA fragmentation and ribosome composition, were not fully comprehended until recently 3-5. Using metabolic or radioactive labeling detection, canonical bisulfite sequencing, methylated RNA immunoprecipitation sequencing (MeRIP-seq) and liquid chromatograph mass spectrometer (LC-MS), an increasing number of mapping approaches for m^5^C have been improved and employed to further elucidate the functions of the m^5^C modification 6-9. For example, a newly developed solid-phase method for single RNA molecule sequencing may be used as a solution to the insufficient sensitivity of second-generation sequencing to detect RNA modification 10. As researchers have increasingly recognized the RNA m^5^C modification, extensive target sites were discovered in multiple RNA classes and diverse organisms 11-17, revealing the crucial effect of the RNA m^5^C modification on eukaryotes.

An integrated m^5^C regulatory network has been revealed, and their writers (methyltransferase), demethylases and readers (proteins that specifically bind to methylation sites) have been identified. Earlier studies have detected DNA methylation induced by the human homologous DNA methyltransferase (DNMT) family, including DNMT1, DNMT2 and DNMT3A/3B. Exclusively, a multiomics study revealed that human DNMT2 not only methylates DNA but also a small RNA (aspartic acid transfer RNA) 18. In recent years, a notable methyltransferase family, NOP2/Sun RNA methyltransferases (NSUNs), has been shown to methylate multiple sites in transfer RNA 13, 17, 19. Additionally, some NSUN family members have target sites on mRNA, rRNA, mitochondrial RNA, noncoding RNA and even viral RNA 7, 20-22. For m^5^C demethylation, ten-eleven translocation (TET) genes were initially implicated as tumor suppressors 23, but subsequently determined to mediate oxidation to 5-hydroxymethyl, 5-formyl, and 5-carboxylcytosine (hm^5^C, f^5^C and ca^5^C), followed by the excision of f^5^C or ca^5^C which is probably induced by thymine DNA glycosidase (TDG) in DNA 24. Studies at m^5^C readers and subsequent physiological functions further indicate the complicacy of cascading regulation at the molecular level. The RNA m^5^C modification is involved in a large number of human cancers, including leukemia, lung cancer, gastric cancer, squamous cell carcinomas, hepatocellular carcinoma and gynecologic cancers 25-29. The conceivable demethylase TET-mediated oxidation and absence of DNA 5mC are associated with cancer progression 30-34. In summary, the m^5^C modification and its elimination are closely associated with molecular and cytological processes in diseases and various cancer types, and thus research on the mechanism and clinical application are hotspots.

In this review, we focus on the pivotal mechanisms of RNA m^5^C modifications involved in metastasis, cell migration and invasion, discussing their functions in cellular biological processes and tumor development. Classified by enzyme family, we review the recent progress in studies of m^5^C writers, demethylases and readers to summarize the current knowledge on methylation associated molecular regulation and pathway activation, as well as the reversion of demethylation. We then discuss the clinical advances in the evaluation and prognosis of human cancers.

## Reversible methylation of nucleic acids

### N6-methyladenosine

In earlier studies, the DNA N6-methyladenosine (m^6^A) modification was established as a pivotal modification in prokaryotes but was not detectable in eukaryotes. Importantly, m^6^A modifications have now been identified as closely related to many biological processes and diseases, such as RNA metabolism related gene expression regulation, DNA damage repair, cell development and differentiation, cell cycle, immune response and even cancer progression 5, 35-38. For instance, research on DNA m^6^A in the human genome indicated that specific enzymes induce methylation and demethylation-mediated gene transcription by targeting the AGG motif 39. The well-known m^6^A methyltransferase and demethylase, methyltransferase-like 3 (METTL3) and fat mass and obesity-associated protein (FTO), respectively, are involved in the DNA damage response to ultraviolet irradiation 40. In contrast, as the most abundant RNA modification in eukaryotes, m^6^A plays a crucial role in epigenetic regulation at the transcriptional level. To date, various RNA classes have been verified to be regulated by m^6^A modification, including precursor mRNAs, long noncoding RNAs, ribosomal RNA and microRNAs 41-43. The diversity of modification sites implies multiple biological functions that reveal an intricate regulatory network of m^6^A. Similarly, METTL3-induced m^6^A promotes the mRNA translation of several oncogenes in human acute myeloid leukemia cells, and then mediates cell proliferation, apoptosis and leukemia progression 44. FTO functions as a regulator of poly(A) site and 3' UTR processing and exon splicing in nuclear mRNAs 45.

### 5-methylcytosine

Studies of the DNA 5mC modification have focused on the transformation of the second structure at first. As physiological and pathological effects are being revealed, DNA 5mC-associated methyltransferases are being identified, including DNMT1, DNMT2, DNMT3A and DNMT3B 46. For instance, the structural and biochemical data of the DNMT1-DNA complex indicate the methylation maintenance function in methylase-induced DNA methylation 47. Otherwise, DNMT1 translocates to the mitochondrial matrix and binds to mitochondrial DNA, which is associated with asymmetric regulation of the translation of heavy chains and light chains in mtDNA 48. In the nucleus, a specific complex composed of DNMT3A or 3B and nucleosomes stabilizes free DNMT3A/3B enzymes, protects them from elimination and promotes further DNA methylation 49. Interestingly, DNMT2 was confirmed to bind DNA in human eukaryotic cells 50. However, according to whole-genome bisulfite sequencing, DNMT2-involved genome modification lacked detectable DNA methylation patterns in a large number of eukaryotes 51. In addition, a human homologue of this methylase, tRNA aspartic acid methyltransferase 1 (TRDMT1), has been reported to share a sequence and function similar to DNMT2 in mediating tRNA m^5^C. We view these two molecules as one RNA m^5^C methylase, which is accepted by mainstream academics.^18^, ^52^. Meanwhile, the NSUN family has been revealed to mediate m^5^C modification in different RNA classes, including tRNA, mRNA, rRNA, vault RNA and enhancer RNA 14, 17, 53-55. The main targets of NSUNs are the 3' untranslated region of mRNA and various cytosines of tRNA. As the most well-known NSUN family menber, NSUN2 (yeast homologue TRM4) participates in cell cycle progression and tumor growth, probably by targeting at known C34, 48, 49, 50 of tRNA in a range of tumors, such as human squamous cell carcinoma and breast cancer 56, 57. While other NSUNs have a variety of functions at post-transcriptional level 11, 14, 22, 29, 58-60.

### N1-methyladenosine

For the N1-methyladenosine (m^1^A) modification, related functional research has become more common in the last five years. The biological effect of the reversible m^1^A modification is to meditate RNA stability and RNA processing, and this modification has been verified to have a transcriptome-wide distribution in mammalian cells and tissues 61, 62. According to recent studies, m^1^A is distributed in mRNAs and enriched at translation start sites (5' UTRs), which may promote translation 63, 64. In addition to the many target sites in mRNAs, m^1^A also occurs in several regions of tRNAs and mitochondrial genes and regulates corresponding biological processes 65. Nevertheless, as an essential component of epigenetic modifications, the biological functions of m^1^A remain ambiguous. The m^1^A level in mitochondrial tRNAs is significantly altered in various human cancers, and is associated with the clinical prognosis 66. For example, by catalyzing the demethylation of m^1^A in tRNA, alkB homolog 1 (ALKBH1) facilitates cancer progression and migration in vivo, resulting in demethylation-induced tRNA fragmentation 67. In summary, the clinical relevance of the RNA m^1^A modification remains unclear and requires further identification.

## m^5^C writers, demethylases and readers

### Methyltransferases mediating the RNA m^5^C modification

**Figure 1:**
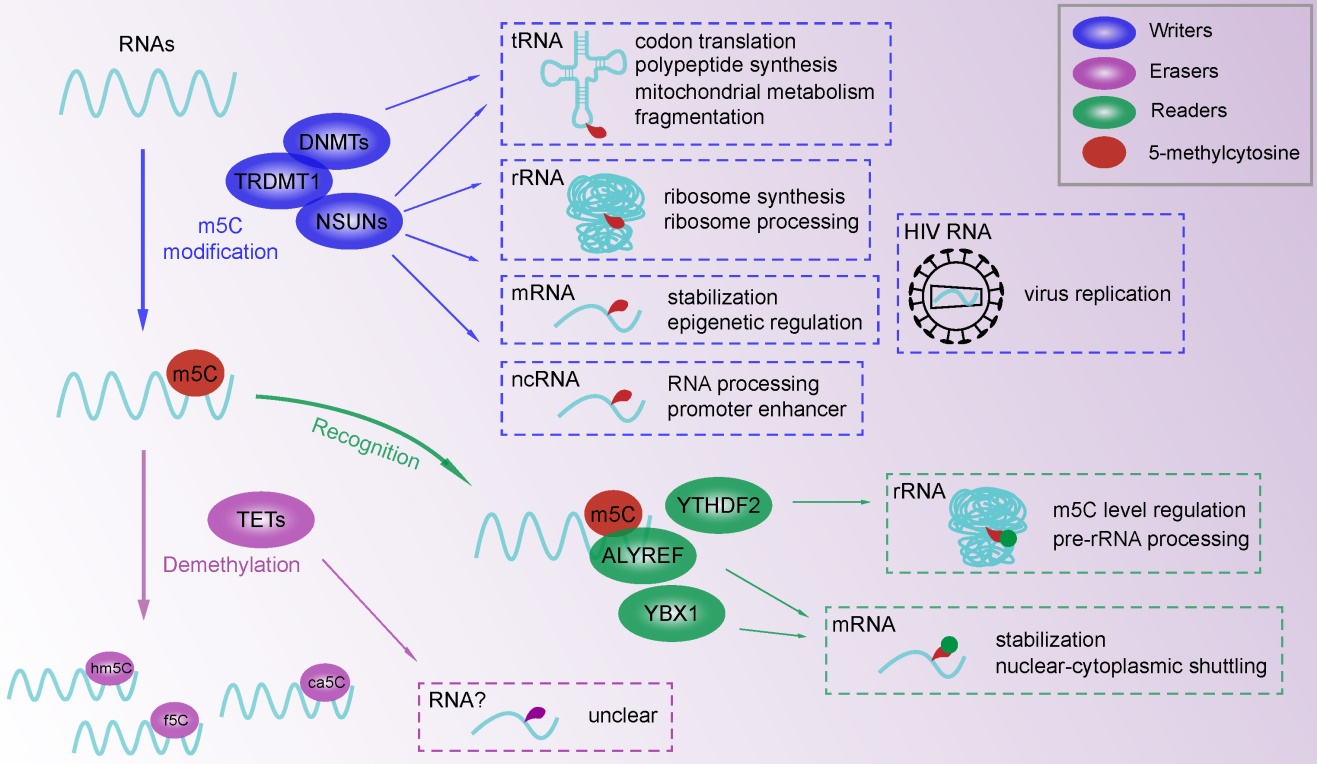
RNA m^5^C modifications with functions of m^5^C writers and readers. M^5^C methylation in tRNA, rRNA, mRNA and ncRNA is involved in RNA processing and metabolism. Binding proteins (YTHDF2, ALYREF, YBX1) for m^5^C modification take part in m^5^C modification in methylase-dependent or independent way. A multicomponent regulatory network is constructed to affect epigenetic regulation.

The formation of m^5^C indicates the installation of a methyl group on the fifth carbon of cytosine in CpG islands, which is regulated by m^5^C methyltransferases and demethylases. The so-called m^5^C “writer” typically possesses highly conserved functional regions that are present in other members of the enzyme family. Numerous m^5^C writers participate in the methylation of extensive RNAs and epigenetic regulation (Figure 1). DNMT2 proteins contain a DNMT family conserved region in the target recognition domain, which shares the DNA-MTase catalytic triad to methylate tRNA substrates 68. An in vitro RNA electrophoretic mobility shift assay confirmed that DNMT2 preferentially binds specific mRNAs other than DNA 69. A similar pattern of double-substrate targeting has been observed for other epigenetic modifications, such as m^6^A, hm^5^C and m^1^A 70-75. To date, 7 human NSUN variants have been identified that recognize RNAs through aspartate side chains in motif VI and target at specific cytosines 76. Despite sharing conserved functional sites with other family members, NSUN2 significantly upregulates the abundance of m^5^C in mRNAs rather than in total RNAs 7. In addition to mRNA methylation, NSUN2-induced m^5^C installation in tRNAs and vault RNAs has also been verified 15, 21, 77. In eukaryotic cells, NSUN1 (yeast homologue NOP2), NSUN4 and NSUN5 (yeast homologue RCM1) target 25S and 28S rRNAs 11, 14, 22. NSUN3 and NSUN6 individually methylate cytosine 34 of mitochondrial tRNA and cytosine 72 of cytoplasmic tRNA, which improves their stability 60, 78. These targeting sites at positions 2278 and 2870 in 25S rRNA and position 3782 in 28S rRNA are in highly conserved ribosome regions, suggesting the functional importance of rRNA m^5^C modification 79. Unlike mRNA methylation, m^5^C enrichment in tRNAs is a structure-specific modification. Targeting sites are mostly located in the T stem loop at positions 48-50 and anticodon loop positions 34 and 38. Sites have also been reported at position 72 of the acceptor arm and at the D stem loop. NSUN7 interacts with promoter-derived enhancer RNAs to indirectly regulate gene expression indirectly 55. The function of NSUN proteins requires an RNA-recognition motif and Rossman-fold catalytic core, which is slightly different from the catalytic site of DNMT2 80. Furthermore, a novel cofactor mechanism is essential for the NSUN-mediated m^5^C modification, such as the S-adenosylmethionine induced methyl transfer process 81.

### m^5^C reversion and the TET family

The oxidation of m^5^C to hm^5^C, f^5^C and ca^5^C is an essential epigenetic modification in eukaryotes. TET protein-mediated demethylation of DNA 5mC was verified in early studies 82, 83. Notably, the appellation of “5-methylcytosine eraser” for TETs is not widely accepted because the essence of TET induced demethylation is to replace the modification by facilitating subsequent oxidations. The oxidation cascade relies on the conserved catalytic core at the carboxyl terminus, which depends on a cofactor. A recent study revealed that reduced iron and α-ketoglutarate are recruited by the double-stranded β‑helix domain in the TET catalytic region for oxidation 84. Interestingly, human TETs prefer hydroxyl and formyl depositions, suggesting a substrate-specific feature 85. Three influential mechanisms for demethylation of the 5mC oxidized base have been proposed: direct removal of the oxidized methyl group, a passive replication-dependent dilution process, and DNA repair-associated excision of modified nucleotides. Active removal is mediated by TDG coupled with base excision repair, which is specific to the formation of f^5^C and ca^5^C 86, 87. In addition, passive degradation of oxidized substrates occurs frequently during persistent DNA replication. This particular process was reported to be related to 5mC demethylation 88. Finally, base excision repair-induced removal of 5mC residues has been widely accepted, although the detailed mechanism of accurate residue elimination has not been elucidated. In addition, non-canonical DNA mismatch repair-induced removal of alkylated and oxidized nucleotides is one of the probable pathways mediating active DNA demethylation 89.

Recently, TET protein-induced RNA hm^5^C modification has been reported in specific eukaryotic cells, including mouse embryonic stem cells, bone marrow mononuclear cells, *Drosophila* and mouse brain cells 70, 75, 90, 91. For example, the distribution and enrichment of RNA hm^5^C in mouse brain tissues was verified by a dot blot analysis, and this modification is associated with Parkinson's disease. In human tissues and cells, a low level of RNA hm^5^C was detected using improved LC-MS/MS methods 92, 93. Researches have not clearly determined whether hm^5^C modifications in RNA and DNA use the same mechanism and regulatory patterns. A recent study suggested that isocitrate dehydrogenases block TET-induced oxidative activity in both DNA and mRNA based on the results of an LC-MS/MS analysis 94. Hence, further study at the molecular level is needed to assess the functional pattern of RNA hm^5^C. However, few mature sequencing methods at single-base resolution are available for oxidized m^5^C mapping, and thus the study of the mechanism of RNA m^5^C demethylation becomes quite challenging. Immunoprecipitation is an RNA-friendly sequencing method to further reveal the elusive biological functions of m^5^C oxidation and demethylation 90. Moreover, a novel bisulfite-free and high-resolution method uses peroxotungstate to conduct the hm^5^C-to-T transition, following cDNA synthesis and base-resolution sequencing 9, which is helpful to comprehensively identify the hm^5^C distribution and function.

### Proteins binding to RNA m^5^C sites

After the m^5^C modification of RNA, several proteins specifically bind to modified sites, leading to the subsequent regulation of biological processes (Figure 1). We named these particular proteins “readers” due to their capability of recognizing m^5^C-containing oligonucleotides. According to a recent study, the m^6^A binding protein, YTH domain-containing family 2 (YTHDF2), shares a conserved residue at the hydrophobic pocket for binding m^5^C-modified RNA 95. Specifically, Aly/REF export factor (ALYREF) displayed a significantly enhanced binding ability to m^5^C-modified mRNAs across the cytoplasm and nucleus, which was confirmed by ALYREF protein sequencing 7. Found in zebrafish early embryos, Y-box binding protein 1 (YBX1), a well-known multifunctional DNA and RNA binding protein , can recognize and bind m^5^C modified mRNAs by residue Trp45 in the cold shock domain of YBX1 96, 97. Overall, the m^5^C regulatory network mainly consists of functional readers and downstream effector, which needs further investigation of novel specific binding proteins.

## Biological function of m^5^C in eukaryote and disease

The m^5^C modification is widely distributed in RNA involved in various biological processes. As a fundamental function of the RNA m^5^C modification, the regulation of RNA function and metabolism is crucial to the development of biological processes (Figure 1). With improved detection approaches for RNA methylation assays, more types of RNA have been identified as m^5^C targets. The RNA m^5^C modification induced by NSUN1 (yeast homologue Nop2) and NSUN5 (yeast homologue Rcm1) regulate ribosome synthesis and processing 11, 14. NSUN4 even participates in mitochondrial ribosome biogenesis 29, 58. The processing of mature RNA is also associated with m^5^C methylation in mRNAs and noncoding RNAs (ncRNAs). For example, DNMT2 is responsible for RNA processing by relocating and interacting with mRNA as a component of the processing complex 98. The processing of noncoding vault RNA in human cells is induced by the m^5^C modification at C69, which maintains the cell cycle in progenitor cells 21. The m^5^C modification promotes RNA stabilization, transportation and translation, but not translation termination induced by NSUN6 mediated mRNA methylation to allow RNAs to perform their regular functions 99. For instance, NSUN2 targets the 3' untranslated region and stabilizes the mRNA of heparin binding growth factor, which regulates cancer progression 54. The reader YBX1 recognizes and binds m5C-modified mRNAs at Trp45 to enhance mRNA stability and early embryogenesis in zebrafish 96. For nucleus-to-cytoplasm transportation, the reader ALYREF interacts with NSUN2-methylated mRNA to mediate shuttling and subsequent translation in the cytoplasm 100. In addition, protein translation and polypeptide synthesis involving tRNAs are regulated by DNMT2-induced m^5^C of the anticodon stem-loop 12, 77, 101. The RNA m^5^C modification is involved in epigenetic regulation by affecting RNA metabolism. RNA fragmentation is a common metabolic pattern of tRNA that is related to epigenetic regulation and cancer progression 102. DNMT2 and NSUN2 were reported to be associated with the biogenesis of tRNA-derived noncoding fragments by altering tRNA fragmentation 103, 104. In addition, NSUN3 targets mitochondrial tRNA, which may be responsible for energy metabolism and protein synthesis 22, 59, 60.

Since RNA function and epigenetic regulation are strongly associated with the occurrence and development of diseases, the role of the RNA m^5^C modification in diseases and cancer is noteworthy. The disorder of tRNA fragmentation induced by DNMT2 leads to high-fat-diet-induced metabolic disturbance in sperm cells 103. The accumulation of tRNA-derived small RNA fragments due to NSUN2 mutation and the absence of tRNA m^5^C leads to reduced protein translation rates, which activates stress pathways and promotes neuronal apoptosis 105. NSUN2 also targets tRNAs in Purkinje cells and neuroepithelial stem cells, which regulate cell replication, differentiation, migration and neurocognitive development 19, 106. NSUN5 was reported to be a promoter of radial glial cell migration and cerebral cortex development 107. Based on these findings, the RNA m^5^C modification is strongly correlated with dysplasia and dysfunction of the nervous system. Diseases related to energy metabolism disorder are also associated with the mitochondrial RNA m^5^C modification. For example, NSUN3 may be associated with multisystem mitochondrial disease associated with a combined oxidative phosphorylation deficiency 108. In leukemia cells, NSUN3/DNMT2 and NSUN1 regulate the formation of 5-azacitidine-sensitive chromatin structures in an antagonistic manners 25. Additionally, the canonical DNA demethylase TET2 was reported to mediate mRNA m^5^C that leads to myelopoiesis in infections, such as sepsis and parasitosis, in the mammalian system 91.

The RNA m^5^C modification in malignant solid tumors has been a focus of cancer research at the epigenetic level. Various m^5^C writers and readers play crucial roles in cancer progression, including cell proliferation, differentiation, migration, invasion and drug sensitivity. Taking the typical m^5^C methylase NSUN2 as an example, NSUN2 has been shown to participate in multiple pan-cancer pathways: promoting cell progression, migration and invasion in breast cancer 57, 109; promoting tumorigenesis and development in skin cancer 27; promoting cell growth, tumor progression and metastasis in urothelial carcinoma of the bladder 54; promoting tumorigenesis and cell proliferation in gall bladder cancer 110; increasing the sensitivity of HeLa cells to 5-fluorouracil 28; promoting cell migration, invasion and drug resistance in esophageal squamous cell carcinoma (ESCC) 111; promoting proliferation in gastric cancer 112; promoting tumorigenesis, migration and invasion in hepatocellular carcinoma (HCC) 113. NSUN4, NSUN5, NSUN6 and NSUN7 are associated with cancer development in HCC, head and neck squamous cell carcinoma (HNSCC), pancreatic cancer and glioma 29, 114-116. In addition, the tumor immune microenvironment is affected by NSUN2 and NSUN6 through multiple pathways, such as the regulation of RNA metabolism, the cell cycle and immune cell activation 117. In addition to the NSUN family-induced RNA m^5^C modification in cancer, DNMT1 overexpression directly leads to hypermethylation of tumor suppressor genes, which results in lung tumorigenesis and a poor prognosis 26. Regarding m^5^C readers, ALYREF is associated with tumor progression in patients with glioblastoma and HCC, but regulates glucose metabolism and tumorigenesis in individuals with bladder cancer 29, 118. YBX1 promotes cell growth, tumor progression and metastasis in bladder urothelial carcinoma 54. More importantly, m^5^C readers are associated with cancer development in collaboration with methylases 54, 119. For the TET family, a downregulated RNA hm^5^C level was reported in cancerous tissues compared to adjacent normal tissues in human colorectal carcinoma and hepatocellular carcinoma, suggesting possible anticancer mechanisms 120. In general, upregulated RNA m^5^C components and high levels of the m^5^C modification are significantly correlated with malignancy and a poor prognosis in patients with the cancers mentioned above and some other cancer types, such as head and neck squamous cell carcinoma (HSNCC) and ovarian cancer 27, 121, 122.

In addition to eukaryotes, in RNA virus HIV-1, the transcription and replication of TAR RNA are restricted by NSUN1 induced RNA methylation 20, while the splicing and translation of mRNA are related to NSUN2-induced RNA methylation 123. The efficient prolongation of HIV latency in CD4^+^ T cells suggests a possible treatment for acquired immune deficiency syndrome.

## Functional role of RNA m^5^C in facilitating metastasis

**Figure 2:**
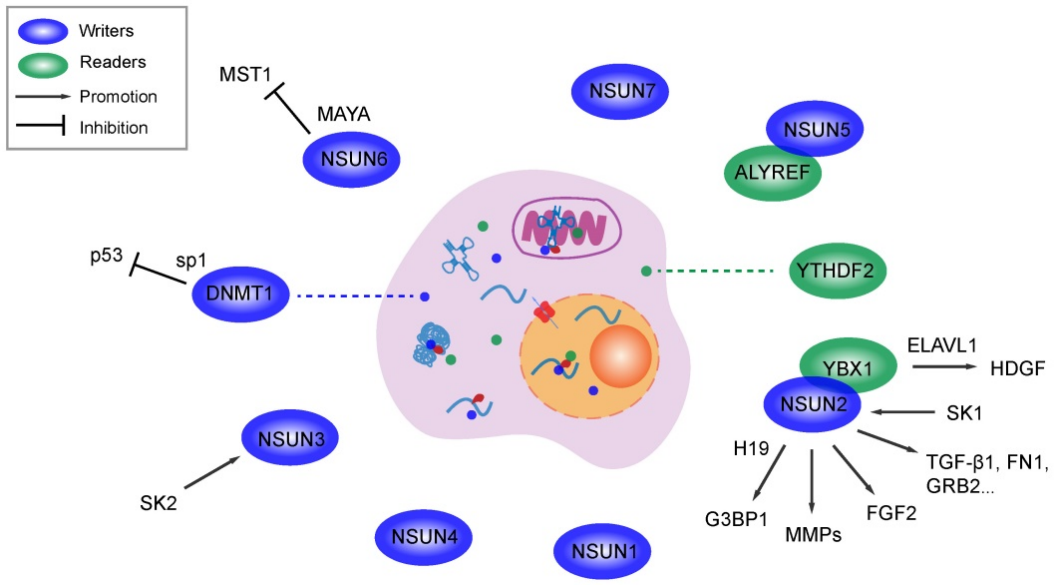
Molecular interactions between m^5^C components and metastasis related genes. M^5^C writers promote metastasis in various cancers by regulate oncogenes or suppressor genes. Several writers and readers are associated with cancer metastasis by unclear mechanism. Methylase and binding protein for m^5^C can work synergistically and promote metastasis.

As the first beneficiary in the field of nucleic acid methylation, m^6^A in RNA possesses multiple epigenetic regulatory activities in various biological processes, diseases and especially cancer 124. With a specific regulator, RNA m^5^C can mediates the activation of oncogenic pathways and forms a microenvironment suitable for the migration and metastasis of various cancer cell lines (Table 1). For instance, NSUN5 and NSUN6 were reported to be associated with metastasis in skin cancer and breast cancer. The former methylase and the specific reader ALYREF are overexpressed in metastatic stage of head and neck squamous cell carcinoma 119. Although the latter participates in RNA-protein interactions, an MST1/2-antagonizing lncRNA for YAP activation inhibits the activity of macrophage stimulating 1 (a protein serine kinase) in an NSUN6-dependent manner, which facilitates bone metastasis in breast cancer 125. In urothelial carcinoma of the bladder, NSUN2 targets the 3' untranslated region (3'-UTR) and stabilizes the mRNA of HGDF by generating the RNA m^5^C modification, while the reader YBX1 binds to the m^5^C region with the help of the partner protein ELAVL1 (an mRNA stability maintainer). The activation of the NSUN2/YBX1/HDGF axis was proven to promote cell growth, tumor progression and metastasis 54. The expression levels of NSUN2 and NSUN3 are downregulated by knocking out proto-oncogenic isozymes (sphingosine kinases 1 and 2, SK1 and SK2) in metastatic prostate cancer and breast cancer. SK1 and SK2 silencing alter the activation of protumorigenic genes relevant to classic membrane signal transduction pathways, such as epidermal growth factor, the EMT, cell cycle, cell motility and DNA stability 126. NSUN2 recognizes and binds to the canonical lncRNA H19 and NMR (NSUN2 methylated lncRNA). In ESCC, methylated NMR interacts with bromodomain PHD finger transcription factor (BPTF) and leads to the overexpression of oncogenes (MMP3 and MMP10) that are involved in cancer cell migration and invasion by activating ERK 1/2 111. Although H19 expression is elevated in various tumors, the lncRNA is stabilized by the NSUN2-induced m^5^C modification, which specifically binds to oncoprotein G3BP1. The mechanism was verified to be associated with tumorigenesis, malignancy and cell migration and invasion. Poor differentiation of cancer cells was observed in H19-overexpressing hepatocellular carcinoma 113. The mRNA encoding autotaxin (ATX) is one target of NSUN2. ATX and its product are famous for exerting multiple membrane functions and mediating cancer development, and activation of the NSUN2/ATX/ALYREF axis promotes mRNA nucleus to cytoplasm transport and cell migration in glioma 127. NSUN2 induced tRNA methylation in neuroepithelial stem cells also mediates differentiation and migration. The underlying mechanism is related to the activation of chemoattractant fibroblast growth factor 2 (FGF2), which facilitates cell maturation and cellular activities 106. Moreover, following the upregulation of NSUN2, the proliferation and migration of HEK 293 cells is enhanced by the overexpression of oncogenes that are associated with the cell cycle, focal adhesion, TGF-β signaling pathway and Notch signaling pathway 52, 128. Taken together, NSUN2 is a notable biomarker for predicting tumor metastasis and the prognosis and a promising therapeutic target in clinic applications. Although NSUN2 overexpression was reported to promote metastasis and cell invasion by promoter methylation 109, these studies explicitly indicate that NSUN family-induced RNA m^5^C modifications play a pivotal role in tumor metastasis and cell motility (Figure 2).

As an immature RNA methylase family, the relationship between the DNMT-induced m^5^C modification and cell mobility or metastasis remains unclear. However these proteins play a supporting role in pathways through which tumor suppressor genes function in a DNA methylation-dependent pattern. For example, DNMT1 activity is increased by high level of specificity protein 1 (SP1)-induced endogenous p53 inactivation in lung cancer, while p53 is now better known for its anticancer effect than as an oncoprotein in various tumors, as it is associated with cell survival, invasion, tumor growth, metastasis, the EMT and multiple pro-oncogenic signaling pathways 129. Analogously, TETs-associated DNA demethylation mediates metastasis in malignancies such as hepatocellular carcinoma and lung cancer 130, 131. The abovementioned effects of these writers, demethylases and readers on DNA 5mC do not refute their potential to mediate metastasis and cell migration. More research on m^5^C modification at the transcriptional level and related oncogenic pathways is needed to improve our knowledge of the epigenetic regulation of tumor metastasis.

## Conclusions

Epigenetic modification is increasing in significance in biological processes. The elucidation of nucleic acid methylation has been reported in recent years. Our review focused on the RNA m^5^C modification in tumor cells and other diseases is inevitably deficient and has the potential for further exploration. The m^5^C modification may not be as popular as sibling methylation m^6^A, but it is still an integral part of epigenetic modification. Abundant writers and readers for RNA m^5^C have been identified, leading to a variety of interactions and regulation of biological processes. Typically, DNMT2 recognizes and methylates tRNA through a highly conserved region that was previously linked to DNA methylation. Along with NSUN2, NSUN3 and NSUN6, these enzymes target tRNAs and participate in tRNA fragmentation and energy metabolism. NSUN1, NSUN4 and NSUN5 preferentially bind rRNA and induce the process of ribosome metabolism. For mRNA, DNMT2 and NSUN2 are the main writers, since mRNA m^5^C is critical for numerous cellular biological processes, such as cell growth, differentiation, movement and RNA modification. Furthermore, NSUN2 and NSUN7 bind to noncoding RNAs that regulate cell metabolism 21, 55, 115. Writers of RNA m^5^C usually work in partnership with readers. YBX1 and ALYREF tend to recognize NSUN-induced methylation, which promotes the transportation and improves the stability of target mRNAs. YTHDF2, on the other hand, binds to m^5^C-modified rRNA at the same conserved residue for the m^6^A modification. Unfortunately, erasers for RNA m^5^C have not been comprehensively identified. Members of the well-known DNA demethylase family TETs trigger cascade oxidations, which may activate the underlying mechanism of RNA demethylation in the m^5^C region. Since a duality of methylation in DNA and RNA was found in m^6^A, m^5^C, hm^5^C and m^1^A modifications, studies aiming to identify whether a co-function of same methylation component induces RNA and DNA methylation or demethylation exists and the related mechanisms and outcomes are needed. Nevertheless, different types of RNA methylation are able to collaborate to regulate downstream targets and modulate the development of diseases. For example, NSUN2-mediated m^5^C collaborates with METTL-mediated m^6^A promote p21 expression, and various types of RNA methylation participate in glioma genesis and progression 132, 133.

RNA m^5^C is relevant to numerous diseases, including energy metabolism disorders, hematopoietic system diseases, viral infection and the most remarkable pathological process, the development of malignancies. The expression of RNA m^5^C writers and readers is significantly correlated with a poor prognosis and the TNM classification of neoplasms. These writers and readers are mainly involved in pathways of tumorigenesis and metastasis in the brain, lung, breast, prostate and others tissue. Many cytokines, such as HDGF, TGF-β, FGF2 and G3BP1, which are validated tumor promoters, participate in cell migration and metastasis induced by the m^5^C modification, indicating a strong correlation between the RNA m^5^C level and mobility of cancer cells. Moreover, some studies discovered high RNA m^5^C levels in circulating tumor cells 134. Since the global amount of m^5^C in blood DNA was identified as a promising marker for cancer prognosis, increased general RNA m^5^C levels may be a novel indicator of metastasis. Nevertheless, a more comprehensive regulatory network for cancer cell transfer requires many mechanistic investigations of epigenetic modifications. RNA m^5^C has the potential to be a unique target for clinical cancer assessment and treatment.

Regarding the existing targets of RNA m^5^C modification, gene therapy is a feasible treatment for RNA m^5^C-induced diseases and cancer development. By modifying the gene sequence of key RNA m^5^C methylases, such as NSUN2, reduced RNA methylation may prevent the occurrence of disease or reverse cancer progression. However, considering the non-specificity of gene therapy, a well-designed inhibitor based on molecular research may effectively reduce the function of a specific RNA m^5^C writer and reverse the development of diseases since inhibitors of m^6^A components are being used in cancer therapy. In addition, the upstream molecules that regulate the functional activity of the RNA m^5^C component may represent an optional treatment for diseases related to specific molecules. For example, aurora kinase B phosphorylates NSUN2 at Ser-139 and reduces RNA methylation levels, suggesting that regulation at the protein level is feasible 135. More research focused on molecular structures and functional pathways is needed to completely understand the effect of the RNA m^5^C modification and exploit it in clinical applications.

However, barriers still exist to further RNA m^5^C research when the final goal is to fully understand the RNA m^5^C modification and translate the findings into the clinic. Regarding the detection and research trends of RNA m^6^A modification, detection methods based on homogeneous techniques are still evolving to obtain better resolution or sensitivity 136. We anticipate that RNA methylation detection is both a limitation in research and a key to breakthrough. Because traditional high-throughput techniques for identifying m^5^C sites are inefficient, computer-based learning techniques that quickly and accurately predict RNA m^5^C modifications are urgently needed. For example, the novel m^5^C detection method, direct RNA sequencing revealed a more complex transcriptome characterization than expected in *Arabidopsis* 137. New methods such as “iRNA-m5C” and “iRNA-m5C_SVM” for RNA m^5^C site detection with good predictive performance will be helpful in structural and functional research on the RNA m^5^C modification 138, 139. However, the immediate application of these new approaches will be a further obstacle in research and clinical translation. Integration of broad data sets and establishment of databases are also effective for improving RNA m^5^C research. Database websites such as “RMBase” integrate a large number of RNA methylation data for review and verification while “RMDisease” and “RMVar” have been applied in RNA modification-related disease research 140-142. Prediction tools such as “PEA-m5C” trained with the forest algorithm predict candidate m^5^C modification sites for functional research 143.

## Authors' contributions

BXZ and ZGZ conceived this study and provided financial and administrative support. QFZ and FRL did most of the data generalization and summarization. WC, HRM and HFL help collecting supplement information. All authors further discussed about data and prepared the panels for tables and figures. QFZ wrote the manuscript. All authors read and approved the final manuscript.

## Availability of data and materials

The researches that support the conclusion of this review have been referenced within this article.

## Figures and Tables

**Table 1:** M^5^C RNA methylation induced cell migration and metastasis

Cell type	M^5^C component	Role	Effects	Refs
Bladder urothelial carcinoma	NSUN2 & YBX1	Writer & reader	NSUN2 stabilize the mRNA of HDGF, while YBX1 bind to m^5^C methylation with a partner protein ELAVL1 and promote cell growth, tumor progression and metastasis	54
Breast cancer	NSUN2	Writer	NSUN2 overexpression promotes metastasis and invasion of breast cancer	109
Breast cancer	NSUN6	Writer	The antagonizing lncRNA for YAP activation inhibits activity of the protein serine kinase MST1 in a NSUN6-dependent way, which facilitates bone metastasis in breast cancer	125
ESCC	NSUN2	Writer	NSUN2 methylated lncRNA bind to chromatin regulator BPTF and promotes MMPs expression through ERK pathway	111
Glioma	NSUN2	Writer	NSUN2 deficiency inhibits glioma cell migration by striking autotaxin and lysophosphatidic acid associated pathway	100
HCC	NSUN2	Writer	NSUN2 methylated H19 bind to oncoprotein G3BP1, which may be associated with tumor genesis, migration and invasion	113
HEK 293 cell	NSUN2	Writer	NSUN2 promote cell proliferation and migration by regulating oncogenes that are associated with cell cycle, focal adhesion, TGF-β signaling pathway and Notch signaling pathway	52, 128
HNSCC	NSUN5 & ALYREF	Writer & reader	ALYREF and NSUN5 are overexpressed in metastasis stage of head and neck squamous cell carcinoma	119
Neuroepithelial stem cells	NSUN2	Writer	NSUN2 mediate FGF2 induced migration and differentiation of neuroepithelial stem cells	106
Prostate and breast cancer	NSUN2	Writer	Expression level of NSUN2 and NSUN3 are regulated by proto-oncogenic isozymes (sphingosine kinases 1 and 2) in prostate metastasis cancer and breast cancer, by a possible mediation of epidermal growth factor associated cell migration	126
Prostate cancer	DNMTs	Writer	DNMT1, DNMT2, and DNMT3 are involeved in lymph node metastases of prostate cancer	144

M^5^C writers and readers function as promoters of metastasis in pan-cancer. ESCC, esophageal squamous cell carcinoma. HCC, hepatocellular carcinoma. HNSCC, head and neck squamous cell carcinoma

## Abbreviations

M^5^C: RNA 5-methylcytosine
5mC: DNA 5-methylcytosine
DNMT: DNA methyltransferase
TRDMT1: tRNA aspartic acid methyltransferase
NSUN: NOP2/Sun RNA methyltransferase
TET: ten-eleven translocation enzyme
YTHDF2: YTH domain-containing family 2
ALYREF: Aly/REF export factor
YBX1: Y-box binding protein 1
hm^5^C: 5-hydroxymethylcytosine
f^5^C: 5-formylcytosine
ca^5^C: 5-carboxylcytosine
HNSCC: head and neck squamous cell carcinoma
ESCC: esophageal squamous cell carcinoma
HCC: hepatocellular carcinoma
NMR: NSUN2 methylated lncRNA
